# Characteristics of patients with meningitis after lumbar epidural steroid injection

**DOI:** 10.1097/MD.0000000000032396

**Published:** 2022-12-23

**Authors:** You-Ri Kang, Tai-Seung Nam, Byeong C. Kim, Jae-Myung Kim, Soo Hyun Cho, Kyung Wook Kang, Kang-Ho Choi, Joon-Tae Kim, Seong-Min Choi, Seung-Han Lee, Man-Seok Park, Myeong-Kyu Kim

**Affiliations:** a Department of Neurology, Chonnam National University Hospital, Gwangju, South Korea; b Department of Neurology, Chonnam National University Medical School, Gwangju, South Korea; c Department of Neurology, Chonnam National University Hwasun Hospital, Jeollanam-do, South Korea.

**Keywords:** complications, epidural injection, meningitis, pneumocephalus, steroids

## Abstract

To investigate the clinical, laboratory, and radiological features of meningitis after lumbar epidural steroid injection (M-ESI) without accompanying spinal infection, data of patients with meningitis admitted between January 2014 and December 2021 in a single center were retrospectively reviewed. Among them, patients with a recent history of lumbar ESI were identified, and their medical records were collected. Patients with concomitant infections other than meningitis, including spinal epidural abscess, were excluded. Seven patients with M-ESI were identified. All patients presented with headache and fever without focal neurological deficits, and headache developed shortly after a procedure (median, 4 hours). Cerebrospinal fluid (CSF) analysis showed neutrophilic pleocytosis (median, 6729/μL), elevated protein level (median, 379.1 mg/dL), decreased ratio of CSF glucose to serum glucose (median, 0.29), and elevated lactate level (median, 8.64 mmol/L). Serum level of C-reactive protein was elevated in 6, but serum procalcitonin level was within normal range. No causative pathogen was identified in the microbiological studies. The most frequent radiologic feature was sulcal hyperintensity on fluid-attenuated inversion recovery images (57%), followed by pneumocephalus (43%). Symptoms subsided in a short period (median, 1 day) after initiating treatment with antibiotics and adjuvant intravenous corticosteroids. None of the patients experienced neurological sequelae. Though the cardinal symptoms and CSF findings of M-ESI were comparable to those of bacterial meningitis, M-ESI seems to have distinctive characteristics regarding the clinical course, laboratory parameters, and pneumocephalus.

## 1. Introduction

Acute or chronic low back pain (LBP) is one of the leading causes of seeking medical attention.^[1]^ Lumbar epidural steroid injection (ESI) under fluoroscopy guidance is a widely adopted therapeutic option for LBP since it can be performed easily in an outpatient clinic and is effective in relieving symptoms, thus delaying or replacing surgical management.^[2,3]^ Despite its efficacy, unexpected adverse events such as epidural hematoma, intravascular injections, direct nerve injury, allergic reactions, air embolism, and seizures may occur.^[4,5]^ Careful monitoring and early detection of adverse events after lumbar ESI are important as they could result in life-threatening conditions such as septic shock, albeit rare.^[6,7]^ Still, the adverse events after lumbar ESI tend to be unreported or underestimated.^[4]^

The overall incidence of infectious complications from lumbar ESI, such as soft tissue infection, epidural abscess, osteomyelitis, or meningitis, has been estimated to be around 1 to 2%.^[8]^ Meningitis is often intercurrent with epidural abscess.^[9,10]^ However, a few cases of isolated meningitis after lumbar ESI (M-ESI) in the absence of spinal infection or systemic bacteremia have been reported, which were considered bacterial meningitis (BM) and treated with antibiotics.^[11–13]^ Meanwhile, some researchers suggested that M-ESI might not be infectious but chemically induced as no pathogenic organism was found in the cerebrospinal fluid (CSF) culture.^[14–16]^ As earlier studies have been limited to a small number of case reports, the characteristics of M-ESI remain undetermined. This study aims to investigate the clinical, laboratory, and radiological features of M-ESI.

## 2. Methods

A retrospective review of the dataset of patients with acute meningitis admitted to the neurology clinic in a single center between January 2014 and December 2021 was conducted. Meningitis was defined when the white blood cell count was >5/µL in CSF analysis with the presence of headache, fever, meningismus, or altered mentality.^[17]^ M-ESI was defined when the contracted patient had a recent history of lumber ESI prior to being diagnosed with meningitis. The cases with concomitant infections, such as localized skin and soft tissue infection, epidural abscess, or infectious spondylitis, were excluded. The time of diagnosis of meningitis was defined as when CSF analysis was complete. Demographic data, clinical symptoms and courses, and laboratory and radiological features were investigated. Additional information regarding the procedure of ESI, including the route of injection and delivered drugs, was obtained. This study protocol was approved by the Institutional Review Board at Chonnam National University Hospital (approval number; CNUH-2020-330).

## 3. Results

### 3.1. Demographic information

Out of 810 patients treated with acute meningitis in the period, 7 patients with M-ESI were identified. They consisted of 3 males and 4 females, and the mean age was 69.6. Chronic LBP was the most common reason for the lumbar ESI procedure, except for 1 patient who developed acute pain after a slip-and-fall accident. One patient had undergone lumbar laminectomy 10 years prior, and three patients had repeated ESI without complications. Dexamethasone and local anesthetics, including lidocaine (57.1%), mepivacaine (28.6%), and ropivacaine (14.3%), were delivered, with or without hyaluronidase, through transforaminal (71.4%) or translaminar (28.6%) routes. No one developed complications other than meningitis. Demographic findings of the enrolled subjects are summarized in Table 1.

**Table 1:** Clinical findings of the patients with M-ESI.

	Pt 1	Pt 2	Pt 3	Pt 4	Pt 5	Pt 6	Pt 7
Epidemiology							
Age, yr	83	80	61	50	72	63	77
Sex	F	F	M	M	F	M	F
Medical history	CAD, DL	DL	CAD, HTN	None	DL	DM	Arrhythmia
Onset of LBP	Chronic	Chronic	Chronic	Chronic	Acute	Chronic	Chronic
Hx of spinal surgery	–	+	–	–	–	–	–
Hx of previous ESI	+	+	–	+	–	–	–
ESI to onset, h	3	1	2	4	12	14	6
Onset to diagnosis, h	4	9	3	12	45	25	48
ESI method							
Injected drugs	D/Lid/H	D/Mep	D/Mep/H	D/Lid/H	D/Lid	D/Lid	D/Rop/H
Approach	TF	IL	TF	TF	IL	TF	TF
ESI complications							
Aggravation of LBP	–	–	–	–	–	–	–
Epidural abscess	–	–	–	–	–	–	–
Subcutaneous infection	–	–	–	–	–	–	–
Bleeding complications	–	–	–	–	–	–	–
Motor weakness	–	+	–	–	–	–	–
Sensory symptom	+	–	–	–	–	–	–
Symptoms and signs							
Headache	+	+	+	+	+	+	+
Fever	+	+	+	+	+	+	+
Peak fever, °C	39.2	38.7	38.3	38.6	37.4	37.4	38.1
Nausea/vomiting	–	+	+	–	+	–	–
Neck stiffness	–	+	–	+	–	+	+
Altered mentality	–	+	–	–	+	–	–
Focal neurologic sign	–	–	–	–	–	–	–
Prognosis							
Neurological sequelae	–	–	–	–	–	–	–
Tx to improvement, d	1	1	1	1	1	2	3
Duration of hosp, d	14	16	14	14	15	16	14

– = absence, + = presence, CAD = coronary artery disease, D = dexamethasone, DL = dyslipidemia, DM = diabetes mellitus, ESI = epidural steroid injection, F = female, H = hyaluronidase, hosp = hospitalization, HTN = hypertension, Hx = history, IL = interlaminar, LBP = low back pain, Lid = lidocaine, M = male, Mep = mepivacaine, M-ESI = meningitis after lumbar epidural steroid injection, Pt = patient, Rop = ropivacaine, TF = transforaminal, TX=treatment.

### 3.2. Clinical manifestations

A severe headache with sudden onset accompanied by fever was the initial presenting symptom in all patients. The median time interval from lumbar ESI to the onset of headache was 4 hours (range, 1–14 hours). The time interval from the beginning of symptoms to the diagnosis of meningitis ranged from 3 to 48 hours (median, 12 hours). Two developed mild drowsiness and confusion, but none showed focal neurologic deficits. Just after ESI, 1 patient (Patient 1) developed paresthesia of both legs, and another (Patient 2) experienced transient paraparesis, but both symptoms spontaneously recovered within an hour. Another patient (Patient 6) presented an orthostatic headache that aggravated in the supine position and was initially diagnosed as having a post-dural puncture headache before the diagnosis of meningitis. Clinical features are summarized in Table 1.

### 3.3. Laboratory findings

Blood and CSF samples were collected before the initiation of antibiotics in the 6 patients, except for 1 (Patient 7). In CSF analysis, neutrophilic pleocytosis was found in all patients, and the median value of white blood cell count was 6729/μL (range, 557–15,700/μL). CSF protein levels were elevated in all patients (median, 379.1 mg/dL, range, 89–620 mg/dL). The CSF glucose ratio to serum glucose was decreased (median, 0.29, range, 0.02–0.46). The CSF lactate levels measured in 6 patients were elevated (median, 8.64 mmol/L, range, 3.64–13.2). Polymerase chain reaction for 5 common pathogens (*Haemophilus influenzae*, *Neisseria meningitidis*, *Streptococcus pneumoniae*, *Listeria monocytogenes*, Group B *Streptococcus*), gram staining, and culture did not produce remarkable results. A week later, the follow-up CSF examination was conducted in 1 (Patient 5), and CSF pleocytosis with an elevated protein level was normalized.

In blood analysis, all patients showed leukocytosis (median, 13,700/μL, range, 11,100–20,900/μL). The level of serum C-reactive protein was elevated in 6 (85.7%) and varied from 0.25 to 14.60 mg/dL (median, 2.78 mg/dL; normal level, <0.3 mg/dL). Serum procalcitonin levels were within the normal range (<0.5 ng/mL) in all patients. Serum lactate levels were measured in 4 patients and were elevated slightly in 3 (range, 1.19–2.87 nmol/L; normal level, <2.1 nmol/L). Laboratory findings are summarized in Table 2.

**Table 2:** Laboratory and radiologic findings of the patients with M-ESI.

	Pt 1	Pt 2	Pt 3	Pt 4	Pt 5	Pt 6	Pt 7
CSF analysis							
WBC count, /μL	8614	6251	7583	5425	15,700	7445	557
Neutrophil count, %	90	85	98.6	88.4	90	83	69.4
RBC count, /μL	88	22	50	500	150	160	25
Fresh form, %	97	100	100	100	95	95	95
Protein, mg/dL	507.4	379.1	275.6	264.9	620	500	89
Glucose, mg/dL	63	41	24	45	2	105	43
Glucose ratio (CSF/serum)	0.37	0.27	0.18	0.43	0.02	0.29	0.46
Lactate, nmol/L	8.94	8.64	8.36	5.84	13.2	N/A	3.64
Blood analysis							
WBC count, /μL	12,300	13,700	12,000	13,800	12,300	20,900	11,100
CRP, mg/dL	3.44	5.09	0.25	1.08	14.60	2.78	1.86
Procalcitonin, ng/mL	0.026	0.315	0.020	0.020	0.159	0.403	N/A
Glucose, mg/dL	170	150	130	105	124	361	93
Serum lactate, nmol/L	2.48	2.87	2.8	1.19	N/A	2.5	N/A
Microbiologic studies							
Blood culture	N/G	N/G	N/G	N/G	N/G	N/G	N/G
CSF culture	N/G	N/G	N/G	N/G	N/G	N/G	N/G
CSF gram stain	None	None	None	None	None	None	None
CSF PCR	None	None	None	None	None	None	None
Radiologic findings							
Modality	CT/MRI	CT/MRI	CT/MRI	CT/MRI	CT/MRI	CT/MRI	CT
Pneumocephalus	–	–	+	–	–	+	+
Sulcal FLAIR hyperintensity	+	+	–	+	–	+	N/A
Leptomeningeal ENH	–	+	–	+	–	+	N/A
Pachymeningeal ENH	+	–	–	–	–	+	N/A
Ventriculitis	–	–	–	–	–	+	–
Restricted diffusion	–	–	–	–	–	–	N/A

CRP = C-reactive protein, CSF = cerebrospinal fluid, CT = computed tomography, ENH = enhancement, FLAIR = fluid-attenuated inversion recovery, M-ESI = meningitis after lumbar epidural steroid injection, MRI = magnetic resonance imaging, N/A = not available, N/G = no growth, PCR = polymerase chain reaction, RBC = red blood cell, WBC = white blood cell.

### 3.4. Radiological findings

Computed tomography of the brain was carried out in all patients before the diagnostic lumbar puncture. Pneumocephalus was observed within the basal cistern or subarachnoid space in 3 (42.9%) patients. None of the patients showed intracranial hemorrhage. Gadolinium-enhanced magnetic resonance imaging (MRI) of the brain was performed in all patients except 1, who was not eligible for MRI owing to a cardiac pacemaker. The most common radiologic finding was sulcal hyperintensity (57%) on fluid-attenuated inversion recovery images. Leptomeningeal and pachymeningeal enhancement was observed in 3 (42.9%) and 2 (28.6%) patients, respectively. One (Patient 6) showed straight horizontal fluid-fluid levels within both occipital horns, suggesting ventriculitis. Radiologic findings are summarized in Table 2.

### 3.5. Treatment and prognosis

All patients received empirical combination antibiotics therapy with cephalosporin, vancomycin, or ampicillin under the presumed diagnosis of BM. Six patients (85.7%) received adjuvant corticosteroids along with antibiotics immediately after the diagnosis of meningitis, whose symptoms quite rapidly subsided after starting treatment (median, 1 day). The symptoms of a patient (Patient 7), who did not show clinical improvement with antibiotics therapy for the first 2 days at the other clinic, improved a day after the adjuvant corticosteroid injection. All patients were discharged after 14 days of antibiotic treatment without neurological sequelae.

## 4. Discussion

Although CSF profiles of M-ESI are comparable to BM, our patients showed distinctive features regarding disease course: rapid onset and resolution of symptoms. In the incipient stage, it was remarkable that headache developed in such a short space of time after lumbar ESI, in the fastest case, within an hour. It could be associated with the pathogenesis of M-ESI different from community-acquired BM, where the pathogen reaches the subarachnoid space through the bloodstream or the spread of skull infection.^[18]^ Pneumocephalus and orthostatic headache, observed in this study, might help estimate the pathogenesis since they are scarce in non-iatrogenic meningitis.^[19]^ They may suggest an unexpected injury of the spinal dura mater during the procedure of lumbar ESI.^[20,21]^ Thus the damaged spinal dura could provide a shortcut for infectious organisms or chemical agents to enter the subarachnoid space. Moreover, even in the absence of pneumocephalus, 2 patients experienced transient neurological deficits bilaterally and symmetrically in both legs just after the procedure, which might imply chemical irritation within the intrathecal space rather than direct needle injury on the spinal nerves. In the recovery stage, symptoms dramatically subsided within a day after initiating the treatment in most patients, even those who presented ventriculitis on brain MRI. These promising outcomes might be attributed to low bacterial load or the early therapeutic intervention with antibiotics. In addition, it was not until corticosteroids had been added that a patient with sustained symptoms despite antibiotics therapy improved, which infers that anti-inflammatory drugs, including corticosteroids, might play an influential role in managing M-ESI. Nevertheless, it could be a natural course of M-ESI regardless of the therapeutic strategy, considering the earlier reports where the patients fully recovered within several days only with conservative management without antibiotics or corticosteroids.^[15,16]^

In the previous literature on M-ESI, the pathogen was identified in the CSF culture in some cases,^[11,12]^ while others were not.^[13–16]^ In the latter, there has been a doubt on whether M-ESI was indeed a bacterial infection, and so does our cases. Although CSF culture is the gold standard for diagnosing BM, it takes several days to obtain results, and its sensitivity varies depending on the laboratory. Thus, laboratory markers are widely used in clinical practice for differential diagnosis in the early stage of the disease. Generally, serum procalcitonin and CSF lactate have been considered helpful in discriminating bacterial infection from non-bacterial etiologies,^[22,23]^ but research on patients with M-ESI has yet to be conducted. Intriguingly, contradictory laboratory findings were observed in our study; increased lactate level might indicate bacterial infection, whereas serum procalcitonin within the normal range might not. Some studies on meningitis after major craniotomy showed skeptical results about the differentiating value of serum procalcitonin.^[24,25]^ Moreover, considering that the elevation of serum procalcitonin in BM might be due to co-existing infection outside the central nervous system (CNS),^[25]^ the localized inflammation within CNS in M-ESI might not be enough to influence the serum level of procalcitonin promptly, whether it is due to infection or not. Concerning CSF lactate, its production within CNS is primarily attributed to the host cells rather than bacteria.^[26]^ Additionally, its elevation would not necessarily be due to infection, and any situations inducing anaerobic metabolism in the CNS can increase CSF lactate concentration.^[23]^ Given the unintentional dural injury during ESI, inflammation along the spinal meninges around the lumbar region and notable pleocytosis per se might result in lactate elevation. Furthermore, the diagnostic accuracy of CSF lactate could be reduced in the red blood cell-containing samples, as in our cases.^[27,28]^ Considering the above, levels of CSF lactate and serum procalcitonin might not be able to guarantee or exclude bacterial etiology completely in M-ESI, and cautious interpretation would be required.

There are several limitations to this study. Firstly, the number of enrolled subjects is small. Secondly, this is a retrospective study; thus, several clinical or laboratory parameters were missing, and the methods and chemical agents for the lumbar ESI were not unified. Thirdly, this study included patients with a history of lumbar ESI, although M-ESI may occur after cervical or thoracic ESI procedures.

## 5. Conclusion

The initial presentation and CSF profiles of M-ESI were comparable to those of BM, but disease course, pneumocephalus, and serum level of procalcitonin were not. Further research on biological markers to discriminate the genuine bacterial infection in M-ESI would be needed.

## Author contributions

**Conceptualization:** Tai-Seung Nam, Man-Seok Park, Myeong-Kyu Kim.

**Data curation:** You-Ri Kang, Jae-Myung Kim, Soo Hyun Cho, Kyung Wook Kang.

**Formal analysis:** You-Ri Kang, Kang-Ho Choi, Joon-Tae Kim, Seong-Min Choi, Seung-Han Lee.

**Investigation:** You-Ri Kang, Tai-Seung Nam.

**Supervision:** Tai-Seung Nam.

**Visualization:** You-Ri Kang, Tai-Seung Nam.

**Writing – original draft:** You-Ri Kang.

**Writing – review & editing:** Tai-Seung Nam, Byeong C. Kim.
